# Concurrent Validity and Inter-Rater Reliability of the Motor Optimality Score-Revised in a Neonatal Surgical Population

**DOI:** 10.3390/jcm14227953

**Published:** 2025-11-10

**Authors:** Cathryn Crowle, Michelle Jackman, Carly Luke, Annabel Webb, Michelle Juarez, Larissa Korostenski, Katya Zawada, Remy Blatch-Williams, Catherine Morgan

**Affiliations:** 1 Grace Centre for Neonatal Intensive Care, The Children’s Hospital Westmead, Sydney, NSW 2145, Australia; michelle.juarez@health.nsw.gov.au; 2 Faculty of Medicine and Health, The University of Sydney, Sydney, NSW 2050, Australia; 3 John Hunter Children’s Hospital, Newcastle, NSW 2305, Australia; michelle.jackman@cerebralpalsy.org.au (M.J.); larissa.korostenski1@health.nsw.gov.au (L.K.); katya.zawada@health.nsw.gov.au (K.Z.); 4 The Cerebral Palsy Alliance Research Institute, The University of Sydney, Sydney, NSW 2050, Australia; annabel.webb@cerebralpalsy.org.au (A.W.); remy.blatchwilliams@cerebralpalsy.org.au (R.B.-W.); cmorgan@cerebralpalsy.org.au (C.M.); 5 Queensland Cerebral Palsy and Rehabilitation Research Centre, Child Health Research Centre, Faculty of Health, Medicine and Behavioural Sciences, The University of Queensland, Brisbane, QLD 4072, Australia; carly.dickinson@uq.edu.au

**Keywords:** motor optimality score, general movements assessment, concurrent validity, inter-rater reliability

## Abstract

**Objectives**: This study aims to evaluate the concurrent validity and inter-rater reliability and agreement of the Motor Optimality Score-Revised (MOS-R) in infants following major surgery in the neonatal period. **Methods**: A cross-sectional study of 211 term infants (mean GA 37.85 weeks, SD 2.10) with congenital anomalies requiring neonatal surgery assessed the concurrent validity of the MOS-R with the Bayley III and HINE at 3 months. Inter-rater reliability and agreement were determined using Gwet’s Agreement Coefficient (AC_1_), the intraclass correlation coefficient (ICC), and percentage agreement (%). **Results**: There were 209 infants assessed at 11–16 weeks post-term age (mean 13 weeks, SD 1.21), and a very weak correlation was observed between MOS-R and Bayley III for cognition (*p* = 0.02), expressive communication (*p* = 0.04), and gross motor (*p* < 0.001). When the MOS-R was categorised based on optimality, the only association was gross motor (*p* < 0.002). The MOS-R had a very weak correlation with the HINE total score (0.18, *p* < 0.001). The inter-rater reliability for the total MOS-R was substantial (AC_1_ = 0.72). When the MOS-R was categorised as optimal, mildly reduced, moderately reduced, or severely reduced, we found good levels of agreement between raters (AC_1_ = 0.76, 83% agreement). Perfect agreement (AC_1_ = 1.00, 100%) was found for categorising the MOS-R using a predictive cut score for adverse outcomes (<23 vs. ≥23). **Conclusions**: At three months of age, the MOS-R showed weak associations with the HINE and Bayley III, indicating limited concurrent validity. Despite this, all tools offer valuable clinical insights. The inter-rater reliability for the MOS-R was good for categorising the MOS-R based on optimality and excellent when using a predictive cut-off score.

## 1. Introduction

Infants born with congenital anomalies requiring surgery in the neonatal period are at increased risk of adverse neurodevelopmental outcomes, particularly infants born with congenital heart disease. Impairments in motor, language, and cognitive skills are widely recognised [1], with lower scores reported for cognition, motor, and language development on the Bayley Scales of Infant and Toddler Development III (Bayley III) compared to peers [2,3,4]. Infants with congenital heart disease are also more likely to have motor and language impairment than infants born very preterm [5]. Infants born with non-cardiac anomalies requiring surgery in the neonatal period also demonstrate lower scores than healthy infants on developmental assessment at one year of age, with the greatest difference in the area of gross motor development [2]. Developmental coordination disorder and co-occurring behavioural and emotional difficulties at 8 to 9 years of age are also more prevalent in the cardiac surgical and non-cardiac surgical populations [6].

To detect the infants most at risk for these impairments as early as possible, clinicians need to use evidence-based tools. Early developmental screening is important to link infants and families with appropriate early intervention services in a timely fashion. The Prechtl General Movements Assessment (GMA) [7] is a widely recognised tool that is recommended for use in the early detection of cerebral palsy (CP) for infants with newborn-detectable risks [8], in addition to brain MRI and the Hammersmith Infant Neurological Examination (HINE). Whilst there is excellent evidence that the GMA accurately predicts CP [9,10], evidence that the GMA accurately detects other adverse neurodevelopmental outcomes is moderate, showing better specificity than sensitivity [11]. A combination of assessment tools is needed to identify the risk of delays across motor, cognitive, communication, and/or behaviour domains.

There is a growing body of research aiming to identify the most reliable screening tools for infants under 6 months of age. The Motor Optimality Score-Revised (MOS-R) [12] is one tool with emerging evidence to support prediction of the risk of adverse non-CP neurodevelopmental outcomes [13]. The MOS-R allows a semi-quantitative scoring of the GMA video to assess fidgety movements, in addition to an infant’s movement patterns, age-adequacy of movement, postural patterns, and movement character [7,12]. These subcategories are each scored separately to provide a total score out of a possible total of 28. A recent systematic scoping review [14] reported that the MOS-R has been used in various ways in clinical practice: for the prediction of outcomes, as an outcome measure, and to describe the current status of different cohorts. A non-optimal MOS-R has been associated with the subtype and severity of CP, as well as with impairments across language, motor, and cognitive domains. The GMs Trust now offers specific training on the MOS-R via the Advanced B course, and recent studies have reported on the predictive validity of this tool across various populations [15,16,17].

Despite increased awareness of the MOS-R among clinicians, there are few studies reporting on the psychometric properties of this tool in high-risk populations [14]. Inter-rater reproducibility, encompassing both reliability and agreement, is an important test characteristic that guides the appropriate use of a test in both clinical and research contexts, and shapes how we interpret and place confidence in the results. The inter-rater reliability of the MOS-R has recently been reported with varying levels of accuracy, from ‘good to excellent’ reliability in a large population-based sample of low-risk infants (with clinically acceptable agreement) [18], to ‘good’ in a preterm cohort [15], to ‘fair’ in a mixed cohort of infants at risk of CP and/or adverse neurodevelopmental outcomes [17]; however, there have been no specific reports in a neonatal surgical population.

Furthermore, there are no reports on the concurrent validity of the MOS-R with other commonly used assessment tools at this early age, including the HINE [19] and the Bayley III [20]. Previous research in a neonatal surgical population has demonstrated that these three assessment tools were all associated with outcomes at one year of age [21]; however, no data is available on how these tools relate to each other. Evidence of the concurrent validity of the MOS-R with these other tools may support using a ‘hands off’ assessment at this early time point, which could be particularly advantageous for infants who remain on handling precautions following surgery in the neonatal period or who live remotely and could be assessed via telehealth.

This study aimed to describe the concurrent validity of the MOS-R with other commonly used assessment tools at 3–4 months of age, the HINE and the Bayley III, in a large cohort of surgical infants. The secondary aim was to investigate the inter-rater reliability and agreement of the MOS-R in this population.

## 2. Materials and Methods

### 2.1. Study Design and Participants

This single-centre cross-sectional study included a convenience sample of 211 infants born at term age with congenital anomalies requiring surgery in the neonatal period. Infants were admitted to the neonatal intensive care unit (NICU) at a tertiary children’s hospital between March 2016 and Oct 2018. They all required surgery for either cardiac- (e.g., coarctation of the aorta, transposition of the great arteries, hypoplastic left heart syndrome, pulmonary stenosis) or non-cardiac-related conditions (e.g., gastroschisis, congenital diaphragmatic hernia, malrotation, jejunal atresia). Infants were included in this study if they attended the developmental follow-up clinic at 3–4 months of age and completed assessment of the GMA, HINE, and Bayley III. The clinic enrols all infants admitted to the NICU and requiring major surgery, excluding those infants followed at other perinatal centres due to extreme prematurity or infants with an identified chromosomal abnormality with a known course of lifelong disability (e.g., CHARGE, trisomy 21). A subset of this cohort with known developmental outcome data (95 infants) has been previously reported on [21]. The cohort of infants in the present study did not all attend face-to-face follow-up appointments at one year of age due to the COVID-19 pandemic, which restricted on-site assessments during this period.

Ethics approval for this study was obtained through the Sydney Children’s Hospitals Network Human Research Ethics Committee (LNR/12/SCHN/513).

### 2.2. Procedure

Assessment results for the previously administered Bayley III and HINE of included infants were added to a REDCap database. Infant assessments had been completed by Allied Health clinicians trained in the use of the GMA, HINE, and Bayley III as part of standard clinical care. All assessments were performed during the same appointment for each participant at 3 months of age. The GMA videos were retrospectively scored using the MOS-R by seven Advanced trained assessors, all with additional training in the MOS-R. The group included one GMs Trust tutor, three assessors with 4–5 years’ experience using the MOS-R for both clinical and research purposes, and three assessors with 3 years of clinical experience using the MOS-R. All assessors were blinded to infant clinical details and other assessment results, apart from the infant’s post-term age at assessment, which is necessary to apply the age-adequacy component of the MOS-R.

Two assessors scored each de-identified video independently using the MOS-R, and scores were recorded in a Research Electronic Data capture (REDCap) database hosted by the University of Sydney. Different pairings of assessors were used for each set of videos. Assessors used the MOS-R proforma and item descriptors as per the published guidelines (2019) to score each of the five MOS-R subcategories to reach a total optimality score ranging from 5 to 28.

To investigate the concurrent validity of the MOS-R with the Bayley III and HINE, a consensus score was recorded. The pairs of assessors met to reach consensus for total MOS-R and each subsection of the MOS-R. A third assessor was consulted in instances when agreement could not be reached for any of these categories.

Data on head ultrasound and brain MRI (where available) were also collected from the medical record for each infant, as these tools are also recommended in the guidelines for early detection of CP [8].

### 2.3. Statistical Analysis

Statistical analyses were completed using R v4.3.1 [22]. A significance level of *p* < 0.05 was used for all analyses.

Total MOS-R was further classified as optimal (25–28), mildly reduced (20–24), moderately reduced (9–19), or severely reduced (5–8) [12,23] and using a previously identified cut score for the prediction of outcomes in this population (<23 vs. ≥23) [21]. HINE scores were also classified according to prediction of delay in this population, with a previously identified score of HINE < 50 used as predictive of any delay [21]. Interpretation of Bayley III scores was based on test norms using the scaled score for each domain (mean 10, SD 3): normal development, 7–13; mild delay, 1 SD below; at risk, 2 SD below.

Descriptive statistics (mean, SD, median, range) were used to describe the study sample and to report results on the three assessment tools. To establish concurrent validity, we used the consensus score for the MOS-R with pre-recorded assessment scores for the HINE and the Bayley III. A Spearman’s rank correlation coefficient was used to quantify correlation of the MOS-R total score and MOS-R items with the Bayley III domains, the HINE total score, and the HINE subtests. We investigated associations between the total MOS-R score and delay within the Bayley III domains using a Mann–Whitney test. 

The association between MOS-R total score classification levels (optimal, mildly reduced, moderately reduced, and severely reduced) and continuous Bayley III and HINE scores was investigated via analysis of variance and Kruskal–Wallis tests, respectively. The association between MOS-R total score classified according to a predictive cut score (<23 vs. ≥23) and continuous Bayley III scores was investigated using two-sample *t*-tests. Finally, the association between delay within the Bayley III domains or the HINE and each of the two approaches to classifying MOS-R total scores was investigated using chi-squared tests of association and Fisher’s exact tests.

Inter-rater reliability and agreement were determined using Gwet’s Agreement Coefficient (AC_1_), intraclass correlation coefficient (ICC), and percentage agreement (%). Data analysis was completed with fidgety movements included (range 5–28) and with the exclusion of this subcategory score (range 4–16), as the total MOS-R may differ by up to 11 points based on fidgety movements alone. The AC_1_ treats MOS-R as an ordinal variable and was used for all individual-item inter-rater reliability calculations. Inter-rater reliability was also calculated based on age at GMA assessment: <12, 12, 13, 14, or ≥15 weeks. AC_1_ values were interpreted as 0.00 to 0.21 ‘poor’, 0.21 to 0.40 ‘fair’, 0.41 to 0.60 ‘moderate’, 0.61 to 0.80 ‘substantial’, and 0.81 to 1.00 ‘almost perfect’ [24]. ICC was interpreted as 0.75 to 1.00 ‘excellent’, 0.60 to 0.74 ‘good’, 0.40 to 0.59 ‘fair’, and less than 0.40 ‘poor’ reliability [25].

## 3. Results

From the total cohort of 211 infants, 209 infants (mean gestational age 37.85 weeks, SD 2.10) were included in the analysis, based on assessment at 11–16 weeks post-term age (mean 13 weeks, SD 1.21) (see Table 1). Two infants were excluded, as the GMA was unable to be scored due to poor video quality.

Surgical infants scored below the mean according to the test norms for the Bayley III across all domains; however, the mean scores for each subtest were still within ‘average range’. The mean HINE score was 57.64 (SD 5.58), and the median HINE was 58. Almost all infants had normal fidgety movements (*n* = 197, 94%); however, the mean MOS-R demonstrated that the reduced optimality was 21.79 (SD 3.79). There were *n* = 103 (49%) infants who scored equal to or above the cut score of 23 and *n* = 106 who scored below 23 (51%).

The majority of infants had more normal than atypical movements (*n* = 200, 97%); however, an age-adequate movement repertoire was only present in *n* = 48 (23%) infants. Observed postural patterns varied greatly, but almost all infants had an abnormal movement character (*n* = 203, 97%).

There were 60 infants (29%) who had a head ultrasound during their admission to the NICU. Of these, 44 were normal, and 16 were reported to be abnormal. Only 24 infants (11%) had a brain MRI during their admission, 9 reported as normal and 15 as having changes such as intraventricular haemorrhage, infarcts, or hypoxic/ischaemic injury. Of the 15 infants with abnormal brain MRI, none had an optimal MOS-R (25–28), and 9/15 (60%) had a MOS-R below the cut score for the prediction of adverse outcomes in this population (<23). The average HINE score in this group was 55.6 (SD 7.9).

### 3.1. Concurrent Validity

There was a statistically significant but very weak correlation between the total MOS-R and the Bayley III scores for cognition (*p* = 0.02), expressive communication (*p* = 0.04), and gross motor (*p* < 0.001) when scores were analysed as continuous variables (Table 2). When the MOS-R was categorised using the cut score of 23 for prediction of delay (<23 vs. ≥23), there was only an association for the gross motor domain (*p* < 0.001) (Table 3).

When Bayley III scaled scores for each domain were categorised into delay (<7) or no delay (≥7), there were no associations with the MOS-R when the MOS-R was categorised as optimal, mildly reduced, moderately reduced, or severely reduced, apart from the gross motor domain (*p* = 0.002) (Table 4). When the MOS-R was categorised using a cut score of 23, again, the only association was with the gross motor domain (*p* = 0.002) (Table 3).

When analysing the MOS-R domains, age adequacy on the MOS-R was significantly associated with almost all the Bayley III domains, apart from receptive communication; however, the correlation was very weak (<0.2 in all domains). Observed postural patterns were associated with the gross motor domain on the Bayley III. There were no associations between fidgety movements, observed movement patterns, or movement character and the Bayley III domain scaled scores.

The MOS-R total had a very weak but statistically significant correlation with the HINE total score (correlation coefficient 0.18, *p* < 0.001), as well as with all domains of the HINE (Table 2). Fidgety movements on the MOS-R were significantly associated with total HINE, movement quality and quantity, tone, and reflexes and reactions, with the largest correlation between fidgety movements and HINE movement quality and quantity (correlation coefficient 0.35, *p* < 0.001). Observed postural patterns on the MOS-R were associated with total HINE, posture, and reflexes and reactions.

When MOS-R was categorised as optimal, mildly reduced, moderately reduced, or severely reduced, there was an association with total HINE score when categorised using a predictive HINE score of <50 vs. ≥50 (*p* = 0.07) (Table 4).

### 3.2. Inter-Rater Reliability and Agreement

The inter-rater reliability of the total MOS-R was substantial (AC_1_ = 0.72) and remained high when the fidgety movements sub-score was removed from the analysis (AC_1_ = 0.67). Based on the ICC, the overall inter-rater reliability for the total MOS-R was low (ICC 0.39) but slightly better when the fidgety movements sub-score was removed (ICC 0.51). When the MOS-R was categorised as optimal, mildly reduced, moderately reduced, or severely reduced, we found good levels of agreement between raters (AC_1_ = 0.76, 83.6% agreement). Most notably, when the MOS-R was categorised using the cut score for the prediction of adverse outcomes for this population (<23 vs. ≥ 23), agreement was perfect (AC_1_ = 1.00, 100%) (Table 5).

For individual items on the MOS-R, the highest inter-rater agreement was for movement character (96.9%), followed by observed movement patterns (93.5%), and the lowest was for observed postural patterns (67%). The inter-rater reliability for MOS-R items ranged from poor (AC_1_ = 0.29) for observed postural patterns to almost perfect (AC_1_ = 0.97) for movement character (Table 5).

The inter-rater reliability for total MOS-R was similar, regardless of the age at which the GMA was completed: <12, 12, 13, 14, or ≥15 weeks. Gwet’s coefficient ranged from a minimum of 0.66 at 13 weeks (substantial) to a maximum of 0.83 at 12 weeks (almost perfect), with a percentage agreement of 85.6–92.9%.

## 4. Discussion

This paper aimed to report on the psychometric properties of the MOS-R to understand the validity of assessment tools commonly used at an early time point of infant follow-up. Specifically, we investigated the concurrent validity and inter-rater reliability of the MOS-R in a neonatal surgical population. To screen infants at a very young age for a range of neurodevelopmental delays, clinicians often use a combination of tools. Only a small proportion of infants in this group underwent imaging, either by head ultrasound (29%) or brain MRI (11%), which highlights the importance of using other evidence-based assessment tools.

### 4.1. Concurrent Validity

Typical assessment tools used alongside the GMA and MOS-R at 3–4 months of age include the Bayley III and the HINE. Concurrent validity is important for understanding whether tools provide consistent or different information, which can guide the use of these tools in clinical practice.

Previous research in a surgical population has demonstrated that although the Bayley III, HINE, and MOS-R are all associated with outcomes at one year of age, combining the assessments at one time point did not increase the predictive value [21]. The results of the current study found that these tools lack strong concurrent validity, suggesting that they assess different constructs. The HINE is primarily a neurological assessment, the MOS-R considers an infant’s spontaneous movement and posture, and the Bayley III predominantly reports on the acquisition of developmental milestones that are elicited from an infant using standardised test items.

In this surgical cohort, at 3–4 months of age, there were no strong associations between the assessment tools, despite some statistically significant results. The MOS-R total score and the subcategory of observed postural patterns were associated with the gross motor domain of the Bayley III, and the MOS-R total score was weakly associated with the HINE total score and all HINE domains.

We also found a weak association between HINE scores and MOS-R categorical scores. Not surprisingly, fidgety movements on the MOS-R were associated with movement quality and quantity, tone, and reflexes and reactions on the HINE. Also, as we would expect, observed postural patterns on the MOS-R were associated with posture on the HINE, in addition to reflexes and reactions.

The MOS-R, HINE, and Bayley III assess different components of infant neurodevelopment; therefore, the choice of which tool to use to screen for adverse neurodevelopmental outcomes (not CP) at this young age is multifaceted. It may be based on clinician training, the availability of assessment kits, whether families are able to attend face-to-face appointments as opposed to virtual care, and the time allocated for scheduled appointments. All three assessment tools provide useful information to guide clinical decision-making, despite low predictive ability when used in isolation or when combined. Understanding the specific constructs explored by early assessment tools can be helpful in enabling clinicians, such as occupational therapists and physiotherapists, to provide supports and targeted intervention strategies to support skills across all developmental domains.

### 4.2. Inter-Rater Reliability

Our study has reported a lower overall inter-rater reliability based on the ICC (0.39) than some previously published studies (ICC > 0.86) [15,18,26]; however, it was similar to a recent report in a mixed high-risk cohort of infants (ICC 0.56) [17]. Consistent with other studies, reliability was higher when reported using AC_1_ (0.72) [18] and when ‘fidgety movements’ were excluded from the total score [17]. The AC_1_ is a more appropriate statistic to use when the prevalence of a condition is low (i.e., the small number of infants with absent fidgety movements), as AC_1_ adjusts for chance agreement more appropriately than the ICC [27].

We suggest that the reasons for our lower inter-rater reliability may be multifactorial. The assessors in this study were trained by different tutors, with subtle differences in the interpretation of scoring for some movement and postural patterns. Another factor may be the varying years of experience (ranging from 3 years up to GMs Tutor) and practice frequency in using the MOS-R. The study population comprised complex surgical infants with less typical presentations than other cohorts such as the preterm group. Furthermore, many infants (76%) in this cohort were assessed to have a mildly reduced MOS-R. Previous studies have reported lower inter-rater reliability in the mid-range scores [17,28].

This study reported good levels of agreement between raters for categorising the MOS-R as optimal, mildly reduced, moderately reduced, or severely reduced and perfect agreement when the MOS-R cut score for the prediction of adverse outcomes in this population was used. This is a positive finding and may be more clinically significant than the number of points of difference between raters because the MOS-R category may have the potential to guide clinical pathways. Although the significance of the MOS-R categories is not yet clear, studies have consistently found that higher-risk populations, who are at greater risk of adverse neurodevelopmental outcomes, are more likely to have total MOS-R scores in the sub-optimal range compared to healthy term infants, who are likely to score in the optimal range [18,29]. Further exploration of the clinical significance of these categories, alongside other predictive factors, will be helpful in guiding the use of the MOS-R in clinical practice.

Inter-rater reliability was similarly high in relation to the age at which the GMA was completed, which suggests that infant age at assessment does not impact the reproducibility of the MOS-R. This is consistent with other studies that found high reliability and similar reproducibility across assessment age groups [17,26]. This is important because many factors impact when families are available to attend scheduled follow-up or complete a GMA video at home.

The reliability of the scoring of the MOS-R by clinicians in practice may be improved by a more detailed manual with clarification around the scoring of an infant’s movement repertoire, in particular, items in the observed movement patterns domain that influence age adequacy, e.g., what specifically constitutes “normal” foot-to-foot contact and leg lift. The domain with the lowest level of inter-rater agreement for this study was observed postural patterns, as items such as the variability of finger postures required regular discussion to meet consensus. We recommend that clinicians practice and score the MOS-R as a group and calibrate regularly with others for improved reliability.

The varying length of the GMA video and the impact of infant behavioural state may impact an infant’s opportunity to demonstrate a variety of movement patterns, particularly items that directly relate to the determination of age adequacy, such as ‘foot to foot’. This was not specifically analysed in this study and could be a study limitation. Future research should consider whether shorter GMA videos have fewer movement patterns observed and also whether reproducibility is lower. Research into an infant’s variability in movement repertoire over a day, or even a week, would also be interesting to investigate. Other study limitations include the single-centre design and the lack of generalisability of these results to other infant populations.

## 5. Conclusions

In a group of infants with congenital anomalies requiring early neonatal surgery, the MOS-R showed some associations with the HINE and Bayley III at 3 months of age but overall limited concurrent validity. These tools assess different components of infant neurodevelopment; therefore, using a combination of tools may provide a more thorough picture of an infant’s strengths and weaknesses and help to guide clinical decision-making. The inter-rater reliability and agreement for this population were good when the MOS-R was categorised as optimal, mildly reduced, moderately reduced, or severely reduced and excellent when the MOS-R was categorised using a predictive cut score for adverse outcomes, which has practical and meaningful implications in a clinical context.

## Figures and Tables

**Table 1:** Participant characteristics and assessment results.

*n* = 209	Mean (SD)
Gestational age (weeks)	37.85 (2.10)
Birth weight (grams)	3060 (666.04)
Sex (*n*, %)	Male = 119 (57%)
Cardiac surgery (*n*, %)	124 (59%)
Non-cardiac surgery (*n*, %)	81 (39%)
Both types surgery (*n*, %)	4 (2%)
Age at assessment (weeks)	13 (1.21)
**Bayley III Domain Scores (*n* = 209)**	
Cognition	9.38 (2.42)
Receptive Communication	8.67 (1.55)
Expressive Communication	9.21 (1.37)
Fine Motor	9.76 (2.23)
Gross Motor	8.51 (2.11)
**HINE score (*n* = 209)**	
Total	57.64 (5.58) *Median = 58 (31–68)*
**MOS-R (*n* = 209)**	
MOS-R total	21.79 (3.79) *Median = 22 (7–28)*
Optimal (25–28)	*n* = 31 (15%)
Mildly reduced (20–24)	*n* = 159 (76%)
Moderately reduced (9–19)	*n* = 17 (8%)
Severely reduced (5–8)	*n* = 2 (1%)
MOS-R above predictive cut-off ≥23	*n* = 103 (49%)
MOS-R below predictive cut-off <23	*n* = 106 (51%)
***Fidgety movements***	
Normal	*n* = 197 (94%)
Abnormal	*n* = 0
Absent/sporadic	*n* = 12 (6%)
***Observed movement patterns***	
N > A	*n* = 200 (96%)
N = A	*n* = 7 (3%)
N < A	*n* = 2 (1%)
***Age-adequate movement repertoire***	
Present	*n* = 48 (23%)
Reduced	*n* = 91 (44%)
Absent	*n* = 70 (33%)
***Observed postural patterns***	
N > A	*n* = 87 (42%)
N = A	*n* = 33 (16%)
N < A	*n* = 89 (42%)
***Movement Character***	
Smooth/fluent	*n* = 5 (2%)
Abnormal not CS	*n* = 203 (97%)
Cramped-synchronised	*n* = 1 (1%)

Abbreviations: N = normal; A = atypical; CS = cramped synchronised.

**Table 2:** Association between MOS-R total and Bayley III and MOS-R total and HINE as continuous variables.

	Correlation Coefficient	*p*-Value ^1^
MOS-R Total and Bayley III		
Cognitive	**0.12**	**0.021**
Receptive Communication	0.01	0.848
Expressive Communication	**0.11**	**0.041**
Fine Motor	0.06	0.237
Gross Motor	**0.19**	**<0.001**
MOS-R Total and HINE		
HINE Total	**0.18**	**<0.001**
Cranial Nerves	**0.13**	**0.031**
Posture	**0.16**	**0.002**
Movement Quality and Quantity	**0.15**	**0.010**
Tone	**0.12**	**0.026**
Reflexes and Reactions	**0.12**	**0.027**

^1^ Test of non-zero Spearman’s rho correlation coefficient.

**Table 3:** Association between MOS-R total (based on cut-off score) and categorical Bayley III and HINE scores.

	MOS-R Above Predictive Cut-off ≥23 (*n* = 103)	MOS-R Below Predictive Cut-off <23 (*n* = 106)	*p*-Value
Bayley Cognitive, mean (SD)	9.7 (2.4)	9.1 (2.4)	0.097 ^1^
Bayley Cognitive, *n* (%)			
Delay (<7)	9 (9%)	13 (12%)	0.545 ^2^
No delay (≥7)	94 (91%)	93 (88%)	
Bayley Receptive Communication, mean (SD)	8.7 (1.5)	8.7 (1.6)	0.965 ^1^
Bayley Receptive Communication, *n* (%)			
Delay (<7)	11 (11%)	10 (9%)	0.945 ^2^
No delay (≥7)	92 (89%)	96 (91%)	
Bayley Expressive Communication, mean (SD)	9.3 (1.3)	9.1 (1.4)	0.231 ^1^
Bayley Expressive Communication, *n* (%)			
Delay (<7)	1 (1%)	4 (4%)	0.369 ^3^
No delay (≥7)	101 (99%)	101 (96%)	
Bayley Gross Motor, mean (SD)	9.0 (1.8)	8.0 (2.3)	**<0.001** ^1^
Bayley Gross Motor, *n* (%)			
Delay (<7)	9 (9%)	27 (26%)	**0.002** ^2^
No delay (≥7)	94 (91%)	78 (74%)	
Bayley Fine Motor, mean (SD)	9.9 (2.3)	9.6 (2.2)	0.351 ^1^
Bayley Fine Motor, *n* (%)			
Delay (<7)	7 (7%)	10 (9%)	0.657 ^2^
No delay (≥+)	96 (93%)	96 (91%)	
HINE Total, *n* (%)			
<50 score	6 (6%)	13(12%)	0.168 ^2^
≥50 score	97 (94%)	93 (88%)	

^1^ Two-sample *t*-test; ^2^ Chi-squared test of association; ^3^ Fisher’s exact test.

**Table 4:** Association between categorical MOS-R (based on optimality) and categorical Bayley III and HINE scores.

	Optimal MOS-R (*n* = 31)	Mildly Reduced MOS-R (*n* = 159)	Moderately Reduced/Severely Reduced MOS-R (*n* = 19)	*p*-Value ^1^
Bayley III				
Cognitive, *n* (%)				
Delay (scaled score <7)	3 (10%)	16 (10%)	3 (16%)	0.675
No delay (scaled score ≥7)	28 (90%)	143 (90%)	16 (84%)	
Receptive Communication, *n* (%)				
Delay (scaled score <7)	4 (13%)	15 (9%)	2 (11%)	0.785
No delay (scaled score ≥7)	27 (87%)	144 (91%)	17 (89%)	
Expressive Communication, *n* (%)				
Delay (scaled score <7)	0 (0%)	3 (2%)	2 (11%)	0.128
No delay (scaled score ≥7)	31 (100%)	154 (98%)	17 (89%)	
Fine Motor, *n* (%)				
Delay (scaled score <7)	2 (6%)	12 (8%)	3 (16%)	0.408
No delay (scaled score ≥7)	29 (94%)	147 (92%)	16 (84%)	
Gross Motor, *n* (%)				
Delay (scaled score <7)	1 (3%)	27 (17%)	8 (42%)	**0.002**
No delay (scaled score ≥7)	30 (97%)	131 (83%)	11 (58%)	
HINE				
HINE Total, *n* (%)				
<50 score	2 (6%)	11 (7%)	6 (32%)	**0.007**
≥50 score	29 (94%)	148 (93%)	14 (68%)	

^1^ Fisher’s exact test.

**Table 5:** Inter-rater reliability and agreement of MOS-R.

Measure	Inter-Rater Reliability AC_1_ (95% CI)	*p*-Value	Percent Agreement
Total MOS-R score	0.72 (0.67, 0.78)	**<0.001**	-
Total MOS-R score without fidgety movements	0.67 (0.63, 0.72)	**<0.001**	-
MOS-R score categories (optimal, mildly reduced, moderately reduced, severely reduced)	0.76 (0.69, 0.82)	**<0.001**	83.6%
MOS-R score above/below predictive cut-off (<23)	1.00 (NA)	NA	100.0%
Fidgety movements	0.89 (0.84, 0.94)	**<0.001**	90.2%
Observed movement patterns	0.93 (0.90, 0.96)	**<0.001**	93.5%
Age-adequate movement repertoire	0.62 (0.45, 0.81)	**<0.001**	83.0%
Observed postural patterns	0.29 (0.17, 0.40)	**<0.001**	67.0%
Movement character	0.97 (0.95, 0.99)	**<0.001**	96.9%

## Data Availability

The original contributions presented in this study are included in this article. Further inquiries can be directed to the corresponding author.

## Abbreviations

The following abbreviations are used in this manuscript:

GMA	General Movements Assessment
MOS-R	Motor Optimality Score-Revised
CP	Cerebral palsy
ICC	Intraclass correlation coefficient
AC_1_	Gwet’s Agreement Coefficient
Bayley III	Bayley Scales of Infant and Toddler Development III
HINE	Hammersmith Infant Neurological Examination
